# The Impact of Household Debt on Children’s Depressive Symptoms: Evidence from China

**DOI:** 10.3390/bs15111530

**Published:** 2025-11-10

**Authors:** Xiaoli Huang, Tingyu Li, Liqiong Lin, Christopher Gan

**Affiliations:** 1 College of Economics and Management, Fujian Agriculture and Forestry University, Fuzhou 350002, China; 2221410004@fafu.edu.cn (X.H.); 3236304037@stu.fafu.edu.cn (T.L.); 000q090051@fafu.edu.cn (L.L.); 2 Department of Financial and Business System, Lincoln University, Christchurch 7647, New Zealand

**Keywords:** household debt, children’s depressive symptoms, parent–child intimacy, parent–child conflict, working hours, marital satisfaction

## Abstract

The sustained accumulation of household debt may pose significant challenges to children’s mental health in China, particularly their depressive symptoms. However, limited research has examined this relationship. Using data from the 2020 and 2022 waves of the China Family Panel Studies, covering 805 respondents, we employed a pooled ordinary least squares (POLS) regression model to investigate the impact of household debt on children’s depressive symptoms. Our results show that higher household debt is associated with an increase in children’s depressive symptoms, primarily driven by housing debt and nonbank-sourced debt. These associations appear to operate through reduced parent–child intimacy and increased parent–child conflict. Notably, the adverse impacts are mitigated when fathers work longer hours to repay debt and when marital satisfaction is higher. Overall, our findings highlight important implications for enhancing child welfare and promoting family financial stability.

## 1. Introduction

Since the 2008 Global Financial Crisis, Chinese household debt has increased rapidly, mirroring trends seen in many emerging economies during this period. According to the Bank for International Settlements (BIS), household debt increased from 5713.69 billion yuan in 2008 to 80,170.21 billion yuan by September 2024, representing an average annual growth rate of 18.03%. By September 2024, China’s household debt-to-GDP ratio reached 60.1%, exceeding the emerging market average of 49.1% and marking an increase of 42.2 percentage points since 2008. This surge has been primarily driven by mortgage debt, which accounted for 58.78% of total household debt as of September 2024.

Additionally, the rapid expansion of internet finance and inclusive finance in China has introduced numerous microcredit products, such as Ant Group’s Huabei and JD Baitiao, which have facilitated private consumption and led to a rapid rise in unsecured and credit debt. However, the recent slowdown in China’s economic growth has dampened household income growth, raising concerns about the sustainability of debt accumulation.

While existing literature has extensively documented the negative effects of debt on households’ economic welfare (Costa-Agmon & Gendel-Guterman, 2025), there is growing concern about the potentially severe risks that debt poses to adults’ depressive symptoms (Breuer & McDermott, 2019; Selenko & Batinic, 2011). Childhood is a critical period for personality formation, cognitive development, and socioemotional growth. Children’s mental health directly influences their future quality of life and their ability to adapt socially. However, the relationship between household debt and children’s depressive symptoms remains unclear and requires further investigation.

This study seeks to address this gap by empirically examining the relationship between household debt and children’s depressive symptoms using data from the 2020 and 2022 waves of the China Family Panel Studies (CFPS). It further explores three potential mechanisms through which debt may affect children’s depressive symptoms: parent–child relationships, parental working hours, and marital satisfaction. In addition, the study analyzes how these effects differ across groups defined by parental education, income levels, and rural–urban residency. Overall, this research provides valuable insights into how household debt influences children’s mental well-being and offers important implications for improving child welfare and promoting family financial stability.

## 2. Hypothesis Development

### 2.1. The Association Between Household Debt and Children’s Depressive Symptoms

The Family Stress Model and Social Stress Theory suggest that resource scarcity and instability create economic pressure, which can lead to increased stress, anxiety, and depression (Pearlin, 1989; Conger et al., 1990, 1994; Dwyer et al., 2011; Berger et al., 2016). Borrowing allows families to access resources to overcome financial hardships, stabilize consumption, purchase luxury goods, and invest in long-term goals (Dynan & Kohn, 2007). According to Berger et al. (2016), appropriate borrowing from reliable financial institutions, with manageable costs and controlled debt levels, can temporarily reduce economic stress by improving material resources. However, poor financial management may worsen economic hardship and intensify stressors over time.

Debt can place significant pressure on borrowers, who may struggle with repayments and face potential legal or financial consequences, adversely affecting their mental health. For children, family adversities generally stem not directly from debt itself, but from how parents respond to economic hardship (Conger et al., 2002). The economic pressure caused by debt can heighten interparental conflict and lead to harsher parenting, thereby heightening the risk of depression in children. Liu et al. (2024) report that mortgage debt, rather than non-mortgage debt, primarily drives the association between household debt and children’s psychological well-being. In contrast, Berger and Houle (2019) find that unsecured debt is linked to greater emotional problems in children, while other forms of debt show no such effect. Unsecured debt, often incurred for immediate consumption, typically carries higher interest rates and fees, which intensify economic burdens. Parents’ negative emotions in response to debt pressure may in turn adversely affect children’s psychological development. Building on these discussions, we infer that household debt may exacerbate children’s depressive symptoms. Accordingly, we propose the following hypothesis:

**Hypothesis** **1.** *Household debt increases children’s depressive symptoms.*

### 2.2. The Channel Through Which Household Debt Affects Children’s Depressive Symptoms

The life course principle of “linked lives” provides a useful lens for understanding how debt assumed by adults influences children’s depressive symptoms. Higher levels of household debt can alter parent–child relationships by reducing intimacy and increasing conflict (Liu et al., 2024). Moreover, the burden of repaying debt places parents under stress. As a result, they may devote less time and energy to parenting, leading to fewer positive interactions with their children and diminished parent–child intimacy (Yan et al., 2024). In addition, financial pressures can subject parents to sustained psychological strain, increasing their risk of depression, anxiety, and apathy. Consequently, parents may struggle to provide adequate emotional care, further weakening parent–child intimacy. In environments characterized by low parent–child intimacy, children may display heightened anxiety, feel greater pressure to be self-reliant and make independent decisions, and face an increased likelihood of mental health problems, including depression and anxiety (Raudino et al., 2013).

At the same time, household debt may place parents under considerable financial pressure, increasing the likelihood of irritable or hostile parent–child interactions (Reading & Reynolds, 2001). Parent–child conflict can directly and negatively affect multiple aspects of children’s development, including cognitive growth, emotion regulation, interpersonal adjustment, and the emergence of behavioral problems. Such conflict may also contribute to adverse emotional outcomes, including depression (Buyse et al., 2011). Based on this, we infer that household debt is associated with reduced parent–child intimacy and heightened parent–child conflict, thereby elevating children’s risk of depression.

Based on these insights, we propose:

**Hypothesis** **2a.** *Household debt is associated with greater depressive symptoms among children, indirectly through reduced parent–child intimacy.*

**Hypothesis** **2b.** *Household debt is associated with greater depressive symptoms among children, indirectly through increased parent–child conflict.*

### 2.3. The Effect of Long Working Hours in the Relation Between Household Debt and Children’s Depressive Symptoms

Financial stress often forces parents to work longer hours, potentially moderating the association between household debt and children’s depressive symptoms. To alleviate financial strain, indebted parents may take on precarious employment or extend their working hours to increase household income (Fleming, 2017). However, such demands frequently reduce the time and energy available for caregiving and family interactions. Gender role theory further clarifies this dynamic, highlighting the traditional intra-household division of labor in which men are more often breadwinners and women homemakers (Gershuny et al., 2005; Stickney & Konrad, 2012). Accordingly, the effects of fathers’ and mothers’ long working hours on the relationship between household debt and children’s depressive symptoms differ by gender role.

Fathers’ long working hours may shape the link between household debt and children’s depressive symptoms. Studies suggest that fathers who work longer hours in the breadwinner role may confer economic benefits that support children’s psychological well-being (Cooksey, 1997; Zick et al., 2001). Improved family financial circumstances can reduce the risk of peer discrimination and help safeguard children’s self-esteem (Acosta, 2011). In addition, easing financial constraints may enable children to participate more fully in school activities, expand peer social interactions, strengthen coping resources for managing negative emotions, and ultimately reduce the risk of depression.

Mothers’ long working hours also play an important role in the relation between debt and depression among children. On one hand, longer maternal working hours may increase household income, reduce debt-repayment pressure, and provide children with greater access to material resources (Chen et al., 2017). These additional resources may foster greater trust and social interaction, both key components of non-cognitive skill development that positively affect psychological well-being (Knack & Keefer, 1997). On the other hand, extended maternal working hours may reduce the time available for childcare, thereby increasing children’s vulnerability to psychological difficulties (Morrill, 2011). The availability of alternative care arrangements, such as grandparental care or formal childcare institutions, can partly mitigate these risks (Hank & Buber, 2009). Thus, the potential benefits of longer maternal working hours may be offset by the adverse consequences of inadequate parental care. We therefore infer that longer maternal working hours do not significantly moderate the association between household debt and children’s depressive symptoms.

We therefore propose the following hypotheses:

**Hypothesis** **3.** *Fathers’ long working hours mitigate the negative impact of household debt on children’s depressive symptoms.*

**Hypothesis** **4.** *Mothers’ long working hours do not mitigate the negative impact of household debt on children’s depressive symptoms.*

### 2.4. The Effect of Marital Satisfaction in the Relation Between Household Debt and Children’s Depressive Symptoms

The quality of the marital relationship may shape how parents respond to stressful events, potentially triggering a chain reaction between household debt and children’s depression. When marital satisfaction is high, parents are more likely to adopt proactive strategies to manage debt-repayment pressures and their associated effects (Kim & Kim, 2009). In such cases, parents respond more sensitively to their children’s needs, and effective parent–child communication increases, fostering a secure family environment. Over time, this is likely to have positive effects on children’s physical and psychological development (Zhou et al., 2022).

By contrast, when marital satisfaction is low and families face economic strain, parents are more likely to engage in harsh or hostile parenting practices (e.g., strict discipline), which can heighten children’s mental health problems and increase their risk of depression (Ngai et al., 2018). Low marital satisfaction may also reduce parents’ emotional self-control under debt-repayment pressure, leading them to adopt negative conflict-resolution strategies such as avoidance, cold violence, or aggression (Zacchilli et al., 2009). These dynamics may intensify children’s emotional insecurity, leaving them in a prolonged state of hypervigilance and physiological arousal. Consequently, children have fewer cognitive resources available for information processing and for regulating their emotions and behaviors effectively. This may impair cognitive development and increase problem behaviors such as avoidance and emotional outbursts. We therefore infer that lower marital satisfaction may exacerbate the positive effects of household debt on children’s depressive symptoms. Based on these insights, we propose:

**Hypothesis** **5.** *Lower marital satisfaction exacerbates the negative effect of household debt on children’s depressive symptoms.*

## 3. Data and Sample

### 3.1. Data Sources

This study utilizes data from the China Family Panel Studies (CFPS), conducted by the Institute of Social Science at Peking University. The CFPS covers 25 provinces, municipalities, and autonomous regions across China and surveys approximately 16,000 households and their members. As a large-scale, nationally representative longitudinal survey, the CFPS adopts a multidisciplinary approach to capture both economic conditions and broader social well-being of Chinese residents.

To analyze children’s depressive symptoms, we use the 2020 and 2022 waves, which contain detailed information on children’s self-reported depression scale. Our analysis focuses on children aged 10 to 15. This age range is a developmentally sensitive period of heightened susceptibility to environmental stressors such as financial strain, consistent with evidence from Beckwith et al. (2024) showing that individuals aged 10 to 19 are more prone to mental health problems. Furthermore, this sample selection is dictated by the scope of the China Family Panel Studies (CFPS), which targets its child questionnaire specifically to respondents aged 10 to 15. We merged the children, adult, and household datasets, then performed data cleaning to remove missing or incomplete records. After this process, the final sample consists of 805 observations.

### 3.2. Variable Definitions and Measurements

Children’s depressive symptoms are measured using the 8-item Center for Epidemiological Studies Depression Scale (CES-D), which has been validated in previous research on children’s depressive symptoms in China (Radloff, 1977; Hann et al., 1999; Man et al., 2021; Miloyan & Eaton, 2021). Each item is rated on a 4-point scale reflecting the frequency of symptoms experienced in the past week: “rarely” (less than 1 day), “sometimes” (1–2 days), “occasionally” (3–4 days), and “most of the time” (5–7 days). These responses are coded from 0 to 3, respectively. The total CES-D 8 score ranges from 0 to 24, with higher scores indicating the higher risk of depressive symptoms.

Household debt is measured as the total outstanding debt, encompassing both housing and non-housing debt. Housing debt refers specifically to housing-related loans, while non-housing debt includes credit card balances, medical loans, business-related debt, and other forms of borrowing. Households obtain debt from various sources, such as banks, relatives, and friends. For analysis, the logarithmic transformation of the total loan balance is used to address skewness and facilitate interpretation.

The parent–child relationship is primarily assessed through two dimensions: intimacy and conflict (Peng, 2023). Parent–child intimacy captures the warmth and positive interaction between parent and child. It is measured using three items from the CFPS questionnaire, such as “How often do you discuss school matters with your child?”, “How often do you require your child to complete his/her homework?”, and “Do your parents know who you play with when you’re not at home?”. Each item is rated on a 5-point scale, and the intimacy score ranges from 0 to 15, with higher scores indicating greater parent–child closeness. In contrast, parent–child conflict reflects hostility and disagreement, measured by the item: “How many times has the child quarrelled with their parents in the past month?”

Father’s or mother’s long working hours indicate that the parent spends extended time at work, thereby reducing the time available for domestic and family activities. A parent is classified as working long hours and assigned a value of 1 if they work more than 44 h per week; otherwise, the value is 0.

Marital satisfaction reflects the quality of the marriage relationship and is measured as the average of both spouses’ self-reported satisfaction scores. In the CFPS survey, respondents were asked, “On a scale from 1 to 5, how satisfied are you with your current marital life?” Responses range from 1 (‘very dissatisfied’) to 5 (‘very satisfied’).

To enhance the robustness of our empirical analysis, we include a set of control variables at the individual, parental, and household levels, following Berger and Houle (2016, 2019) and Liu et al. (2024). Children’s characteristics encompass gender, educational attainment, health status, class rank, and interpersonal relationships. Parental characteristics include possession of a high school diploma and drinking behavior. Household characteristics cover household assets, car ownership, educational expenditures, social interactions, livelihood diversification, and regional location. Detailed definitions of all variables are provided in Table 1.

### 3.3. Descriptive Statistics

Table 1 presents summary statistics for the key variables. The mean CES-D 8 score for children’s depressive symptoms is 4.061. The average household debt is 73,307 RMB, with a maximum of 1,100,000 RMB, approximately 15 times the mean, highlighting substantial variability. Parent–child intimacy averages 10.64, while parent–child conflict averages 1.646. Regarding working hours, 67.3% of mothers and 86.8% of fathers work more than 44 h per week, indicating that fathers generally bear a heavier financial burden as primary earners. The average marital satisfaction score is 4.426.

Among children, 55.2% are male, with an average educational attainment of 6.14 years, suggesting most have completed basic education. On average, children rank between the 26th and 50th percentile in their most recent major exam. The mean score for self-reported interpersonal relationships is 6.948, displaying generally positive social relationships.

For parental characteristics, 37.9% have attained a high school diploma, while 32.8% reported drinking alcohol more than three times per week in the past month. Household assets average 401,155 RMB, and educational expenditures average 6257 RMB. The average livelihood diversification index is 0.190, and approximately 50.8% of families own cars.

## 4. Empirical Strategy

Building on prior research, this study employs pooled ordinary least squares (POLS) regression models to investigate the relationship between household debt and children’s depressive symptoms. The baseline POLS model is specified as follows:

(1) $${Children}_{it}=\alpha_{0}+\alpha_{1}{debt}_{it}+\alpha_{2}{control}_{it}+\varepsilon_{it}$$

where ${Children}_{it}$ represents the depressive symptoms of child *i* at time *t*. The explanatory variable ${debt}_{it}$ denotes the total household debt. ${control}_{it}$ represents control variables, including children’s characteristics, parental characteristics, and household characteristics. Finally, $\varepsilon_{it}$ is the residual error term capturing unobserved factors.

Furthermore, following the approach of Baron and Kenny (1986), we evaluate the mediating role of the parent–child relationship by estimating the following models:

(2) $${Children}_{it}=\alpha_{3}+\beta_{1}{debt}_{it}+\varphi_{1}{control}_{it}+\varepsilon_{it}$$

(3) $$M_{it}=\alpha_{2}+\beta_{2}{debt}_{it}+\varphi_{2}{control}_{it}+\varepsilon_{it}$$

(4) $${Children}_{it}=\alpha_{4}+\beta_{3}{debt}_{it}+\rho_{3}M_{it}+\varphi_{3}{control}_{it}+\varepsilon_{it}$$

where $M_{it}$ represents the two mediating variables measuring the parent–child relationship: parent–child intimacy and parent–child conflict. In Equations (2)–(4), ${Children}_{it}$ is the outcome variable, and ${debt}_{it}$ is the exposure variable. Following the mediation analysis procedures outlined by Baron and Kenny (1986) and VanderWeele (2016), a mediating effect exists if the coefficient $\varphi_{1}$ in Equation (2) and the coefficient $\beta_{2}$ in Equation (3) are both statistically significant under the same covariates. Further, if the coefficient $\beta_{3}$ in Equation (4) is significant but smaller in magnitude than $\beta_{1}$, and the coefficient $\rho_{3}$ is significant, this indicates a partial mediation effect. However, if $\beta_{3}$ in Equation (4) is no longer significant, is smaller than $\beta_{1}$, and $\rho_{3}$ remains significant, this suggests a complete mediation effect. All other model specifications are consistent with the basic model.

To examine the moderating effects of fathers’ and mothers’ long working hours, as well as marital satisfaction, we further specify the following model:

(5) $${Children}_{it}=\alpha_{5}+\gamma_{1}{debt}_{it}+\gamma_{2}{\left(debt\times P\right)}_{it}+\gamma_{3}P_{it}+\gamma_{4}{control}_{it}+\varepsilon_{it}$$

In Equation (5), $P_{it}$ represents the three moderator variables: father’s long working hours, mother’s long working hours, and marital satisfaction, respectively. The term ${\left(debt\times P\right)}_{it}$ denotes the interaction between household debt and each moderator. The coefficients $\gamma_{3}$ capture the direct effects of the moderators $P_{it}$ on children’s depressive symptoms. The coefficient $\gamma_{2}$ on the interaction term reflects the moderated effect, indicating how the relationship between household debt and children’s depressive symptoms varies depending on the levels of parents’ long working hours and marital satisfaction.

## 5. Empirical Results

### 5.1. Benchmark Results

Table 2 presents the results from linear regression models estimating the association between household debt and children’s depressive symptoms. The findings show that higher household debt is significantly associated with depressive symptoms in children. In the simplest model without any control variables, each increase in household debt corresponds to a 3.5% increase in the risk of psychological distress among children. This positive relationship remains robust in column (2), where controls for children’s characteristics (health status, class rank, and interpersonal relationships), parental characteristics (possession of a high school diploma, and drinking behavior), and household characteristics (household assets, car ownership, educational expenditures, social interactions, livelihood diversification, and regional location) are included.

Additional findings from the control variables indicate that improved mental health outcomes are associated with stronger interpersonal relationships. Households with greater assets tend to have children with lower depression scores. Children from families without cars are at greater risk of depression than those from car-owning families. Lower educational expenditure is associated with increased psychological issues in children. Lastly, higher livelihood diversification is positively correlated with children’s depressive symptoms, suggesting that more diversified household income sources relate to better mental well-being.

### 5.2. Robustness Test

The benchmark results in Table 2 demonstrate a significant effect of household debt on children’s depressive symptoms. However, these estimations may be subject to potential endogeneity issues, including measurement errors and sample selection bias. To address these concerns and ensure the reliability of our findings, we employ several robustness checks. Specifically, we use the instrumental variable (IV) approach, propensity score matching (PSM), inverse probability weighted regression adjustment (IPWAR), and alternative variable measurements. These methods help to mitigate potential confounding effects and validate the consistency of the baseline results.

#### 5.2.1. Considering Endogeneity: Estimate Results of Instrumental Variables

Potential endogeneity may arise from simultaneity between household debt and children’s depressive symptoms. Two main issues contribute to this concern:

First is omitted variable bias. For example, adherence to traditional family conservatism could influence both children’s depressive symptoms and parental financial behavior. Failure to account for such unobserved factors could bias the estimated relationship between debt and depressive symptoms.

Second is reverse causality. The higher risk of depressive symptoms in children might increase household expenses or lead parents to accumulate more debt (e.g., medical costs or reduced parental work capacity), which in turn could confound the direction of causality.

To mitigate these endogeneity concerns, we implement an instrumental variable (IV) approach to obtain consistent estimates of the effect of household debt on children’s depressive symptoms.

We employ the instrumental variable (IV) approach to re-estimate the benchmark model, aiming to address potential endogeneity concerns. Specifically, families are grouped by their counties and further categorized into four income percentile groups. For each group, we calculate the average household debt of all other families within the same county and income group. This average debt level serves as the instrumental variable.

The IV satisfies the relevance condition because the average debt of families with similar income levels in the same county captures the local credit environment and consumption behaviors, which influence an individual family’s debt decisions. Thus, the instrument is strongly correlated with the household debt of the family in question.

Regarding the exogeneity condition, the regional average debt of families with similar income level is unlikely to directly affect an individual child’s depressive symptoms, as it reflects aggregate financial conditions rather than specific household circumstances. Therefore, the instrumental variable can be considered approximately exogenous and valid for identifying the causal effect of household debt on children’s depressive symptoms.

Panel A in Table 3 presents the estimation results from the two-stage least squares (2SLS) instrumental variable model that accounts for endogeneity. The coefficient on household debt remains positive and is statistically significant at the 10% level. These findings further reinforce the robustness of the benchmark results, confirming that higher household debt is associated with the higher risk of children’s depressive symptoms even after addressing potential endogeneity concerns.

#### 5.2.2. Considering Sample Selection: Propensity Score Matching (PSM)

The relationship between household debt and children’s depressive symptoms may be influenced by self-selection bias. Families with similar observable characteristics might differ in unobserved factors such as behavioral habits, values, and mindsets, which also affect children’s psychological well-being. To reduce potential biases arising from such sample selection issues, we re-estimated the model using the Propensity Score Matching (PSM) method. This approach matches indebted families with non-indebted families that have similar observable characteristics, allowing for a more reliable estimation of the effect of household debt on children’s depressive symptoms.

Panel B in Table 3 presents the estimation results using the nearest neighbor and kernel matching methods. The 1:2 nearest neighbor matching results show that children from indebted households have a 48.33-percentage-point-higher likelihood of experiencing mental health problems compared to children from non-indebted households, significant at the 5% level. Similarly, the 1:4 nearest neighbor matching indicates that household debt increases the risk of children’s depressive symptoms by 51.93 percentage points, also significant at the 5% level. The results from radius matching and kernel matching are consistent with the nearest neighbor estimates and thus are not reported here. These findings provide additional support for the robustness of our benchmark results.

#### 5.2.3. Considering Sample Selection: Inverse Probability Weighted Regression Adjustment (IPWRA)

Panel C in Table 3 reports the IPWRA estimation results, which assess the effect of household debt on children’s depressive symptoms. The findings indicate that debt significantly increases children’s depressive symptoms. Specifically, the average treatment effect (ATE) shows that, compared with children from non-indebted families, those from indebted families have a 3.88% higher likelihood of experiencing mental health problems. These results further support the robustness of our benchmark findings.

#### 5.2.4. Consider Variables: Replace Explanatory Variable (Debt Ratio)

In the benchmark regressions reported in Table 2, household debt is measured by the absolute amount of debt. However, this measure may not fully capture the debt repayment pressure faced by households. For example, households with higher income or asset levels may sustain larger debts without experiencing severe repayment burdens. To better reflect households’ solvency and debt stress, this study follows prior literature and replaces the debt amount with two alternative indicators: the debt-to-income ratio and the debt-to-asset ratio. Columns (1) and (2) of Table 4 present the Probit model estimation results using these ratios as explanatory variables. The results confirm that both the debt-to-income ratio and debt-to-asset ratio remain significantly associated with increased risk of children’s depressive symptoms.

### 5.3. Heterogeneity Analysis

Different types of household debt can impose varying levels of economic pressure on families. To explore this, we categorize debt into housing debt and non-housing debt based on the debt’s intended purpose. Additionally, debt is divided into bank-sourced debt and nonbank-sourced debt according to its origin. Beyond debt types, we further analyze the heterogeneity of debt effects on children’s depressive symptoms by grouping samples based on key demographic and socioeconomic characteristics: child’s gender, parental educational attainment, household income level, and rural-urban residency. This subgroup analysis aims to uncover whether and how the impact of household debt on children’s depressive symptoms varies across different population segments.

We first estimate the heterogeneous effects of different types of household debt on children’s depressive symptoms. Columns (1) and (2) of Table 5 present the results from POLS regressions with a full set of control variables, examining two specifications of relative debt: housing debt and non-housing debt. The results indicate that housing debt significantly increases children’s psychological distress, while the effect of non-housing debt is not statistically significant. Specifically, a 1% increase in housing debt is associated with a 3.5% increase in children’s depressive symptoms.

We also examine how household debt from different sources influences children’s depressive symptoms. Columns (3) and (4) of Table 5 present the regression results for bank-sourced debt and nonbank-sourced debt, respectively. The findings indicate that a 1% increase in nonbank-sourced debt is associated with an approximately 4.7% rise in children’s depressive symptoms.

The relationship between household debt and children’s depressive symptoms also appears to vary by gender. Panel A in Table 6 indicates that household debt significantly affects girls’ depressive symptoms, while the effect on boys is statistically insignificant.

We also examine the heterogeneous effects of parental education attainment. Panel B of Table 6 presents the estimated effects of household debt on children’s depressive symptoms across different parental education levels. Column (1) reports the results for families in which parents have a primary school education or below, while Columns (2) and (3) present estimates for those with a high school diploma and those with a university degree or higher, respectively. The results reveal that the estimated coefficient of household debt is significantly positive only in the primary school subgroup. Specifically, a 1% increase in household debt is associated with a 4.2% increase in children’s psychological distress in families where parents have low educational attainment. In contrast, the effects in the high school and tertiary education subgroups are statistically insignificant.

We further examine the heterogeneous effects of household income. Panel C of Table 6 reports the regression results across different income groups. Columns (1) and (2) indicate that the coefficient of household debt is significantly positive at the 5% level for low-income households. Specifically, a 1% increase in debt among low-income families is associated with a 6.4% rise in children’s psychological distress. In contrast, the impact of household debt on children’s depressive symptoms is statistically insignificant for higher-income households. These results suggest that children in financially constrained households are more vulnerable to the adverse psychological effects of household indebtedness, likely due to greater financial insecurity and limited coping resources.

We also examine the heterogeneous effects of household debt across urban–rural residency. Panel D of Table 6 reports the estimated impact of debt on children’s depressive symptoms in both settings. According to columns (1) and (2), the results reveal that household debt significantly increases the likelihood of psychological distress among children in rural areas, whereas no statistically significant effect is observed in urban areas. Specifically, within rural households, a 1% increase in debt is associated with a 5.1% rise in the probability of children experiencing psychological problems.

These findings suggest that rural families may face greater challenges in providing adequate parenting resources, such as nutrition, healthcare, education, and recreational opportunities, linking to comparatively lower levels of economic development. This structural disadvantage often necessitates borrowing to meet even basic household needs, thereby increasing financial strain. Under such circumstances, children in rural households may become more susceptible to emotional instability, manifesting as feelings of hopelessness, anxiety, and irritability, all of which can severely impair their psychological well-being.

### 5.4. Mechanism Analysis

Following the established mediation analysis procedures, we first examine the mediating roles of parent–child intimacy and parent–child conflict in the relationship between household debt and children’s depressive symptoms. Initially, we confirm the direct effect of debt on children’s psychological well-being. As shown in columns (1) of Panel A and Panel B of Table 7, a 1% increase in household debt is associated with a 4.7% increase in the risk of children’s psychological distress.

In the second step, we assess the impact of debt on the mediators—parent–child intimacy and parent–child conflict. Columns (2) of Panel A and Panel B indicate that a 1% increase in debt results in a 2.6% decrease in parent–child intimacy and a 5.9% increase in parent–child conflict.

Finally, after including parent–child intimacy and parent–child conflict in the regression models (columns (3) of Panel A and Panel B), the coefficient for household debt diminishes, while the coefficients for the parent–child relationship variables remain statistically significant. This pattern confirms the presence of indirect effects, suggesting that the parent–child relationship partially mediates the effect of household debt on children’s depressive symptoms.

Hence, based on our estimations, we have confirmed the indirect pathways hypothesized in 2a and 2b:

- Debt → Parent–Child Intimacy → Children’s Depressive Symptoms.
- Debt → Parent–Child Conflict → Children’s Depressive Symptoms.

These findings suggest that household debt places significant psychological stress on parents, which may manifest as increased irritability or hostility in parent–child interactions. Such dynamics reduce parent–child intimacy and elevate parent–child conflict, thereby increasing children’s risk of depressive symptoms.

As shown in Table 8, we examine whether the long working hours of the father or mother moderate the relationship between household debt and children’s depressive symptoms. Columns (1) and (2) indicate that the coefficient of debt remains positively significant, while the interaction term between the father’s long working hours and household debt is negatively significant for children’s depressive symptoms. In contrast, the coefficients for the mother’s long working hours, household debt, and their interaction term are statistically insignificant. These results suggest that an increase in the father’s working hours mitigates the negative effect of household debt on children’s depressive symptoms.

To validate this finding, we redefine long working hours as working more than 40 h per week for both parents, and the results remain consistent. These findings provide empirical support for Hypotheses 3 and 4.

Column (5) in Table 8 shows that the coefficient of debt is positively significant, while the interaction term (marital satisfaction × debt) is negatively significant. This finding supports Hypothesis 5, indicating that the effect of household debt on children’s depressive symptoms is moderated by marital satisfaction. In other words, higher levels of marital satisfaction alleviate the negative impact of debt on children’s depressive symptoms.

## 6. Discussion

### 6.1. Effect of Household Debt on Children’s Depressive Symptoms

We investigate the impact of household debt on children’s depressive symptoms using data from the 2020 and 2022 waves of the China Family Panel Studies (CFPS), which covers 805 respondents. A pooled ordinary least squares (POLS) model is employed for the baseline estimation. To robustly verify these results, we apply several methods: the instrumental variable approach, propensity score matching (PSM), inverse probability weighted regression adjustment (IPWRA), and replacement metrics. Our analysis also extends to exploring heterogeneous effects and potential mechanisms. These findings advance the understanding of how household debt affects children’s mental health, highlighting implications for enhancing child well-being and family financial health.

The results confirm that household debt significantly increases children’s depressive symptoms, supporting Hypothesis 1. Our estimation indicates that a 1% increase in household debt is associated with a 4.7% rise in the risk of child psychological distress. This finding aligns with the work of Berger and Houle (2019), who demonstrated that parental responses to financial hardship can exacerbate children’s psychological distress. The relevance of this mechanism is underscored by the context of rapidly accumulating household debt in China, which imposes significant financial and time constraints on parents, thereby acting as a critical stressor on their mental health (Sweet, 2021).

### 6.2. Mechanism of the Effect of Household Debt on Children’s Depressive Symptoms

We found that household debt is associated with higher levels of depressive symptoms among children, and this relationship operates through two key mechanisms: reduced parent–child intimacy and increased parent–child conflict, thereby supporting Hypotheses 2a and 2b. The pressure of debt repayment burdens parents, which can diminish positive parent–child interactions and, simultaneously, heighten conflict and the likelihood of harsh parenting. These adverse family dynamics, in turn, increase children’s vulnerability to depression and anxiety (Liu et al., 2024; Yan et al., 2024; Mills et al., 2013; Raudino et al., 2013).

Our results support Hypotheses 3 and 4, revealing a gendered moderating effect: fathers’ long working hours attenuate the negative impact of debt on children’s depressive symptoms, whereas longer maternal working hours show no significant moderating effect. One possible explanation is that traditional gender roles assign distinct divisions of labor between fathers and mothers. For fathers, the economic benefits of longer hours can improve household finances, easing constraints on children’s social activities and broadening their channels for emotional expression (Acosta, 2011). For mothers, the relationship is more complex. While increased income from longer hours may enhance children’s quality of life and mental health (Chen et al., 2017), it can also reduce time available for childcare (Morrill, 2011). This negative effect, however, can be mitigated by care from grandparents or other elders (Hank & Buber, 2009). Consequently, the positive and negative influences of mothers’ longer working hours appear to offset one another, resulting in a net non-significant effect on the debt-depression link.

Our findings support Hypothesis 5, indicating that lower marital satisfaction exacerbates the negative impact of household debt on children’s depressive symptoms. This aligns with the work of Ngai et al. (2018), suggesting that in contexts of low marital satisfaction, debt-related stress is more likely to manifest in harsh or violent parenting, which in turn leads to increased emotional and behavioral problems in children.

### 6.3. Heterogeneous Effects on Different Groups

Our analysis reveals that household debt is associated with increased depressive symptoms in children, with this association primarily driven by housing debt and nonbank-sourced debt. This finding regarding housing debt contrasts with studies like Berger and Houle (2016), which highlight the role of non-housing debt. Unlike housing debt, non-housing debt is often incurred for immediate consumption, placing short-term financial pressure on parents. This pressure, in turn, can increase children’s depressive symptoms. However, in the Chinese context, household debt is disproportionately concentrated in housing debt, largely due to the rapid escalation of property prices over the past two decades. Moreover, Chinese households typically face higher mortgage interest rates than those in most developed economies, resulting in greater debt-servicing burdens. This financial strain may contribute to deteriorating parental mental health, reducing the quality of caregiving and, in turn, adversely affecting children’s physical and psychological development.

Furthermore, we find that nonbank-sourced debt (e.g., private loans) has a distinct negative effect. Unlike formal bank-sourced loans, nonbank-sourced debt often relies on personal relationships between lenders and borrowers. Consequently, families with such debt may face social criticism, strained relationships with neighbors, or even alienation from friends and relatives. This social exclusion can foster a sense of inferiority or shame in children, particularly in peer interactions, ultimately exacerbating their psychological distress.

Our analysis further reveals that the detrimental impact of household debt on children’s mental health is not uniform but is concentrated in vulnerable subgroups. Socioeconomically, the effects are more severe in families with low parental educational attainment and low income. Consistent with Berger and Houle (2019), we find that less-educated families, who face higher borrowing costs and greater repayment pressure, experience more significant psychological harm to their children. Similarly, in line with Lea (2021), the debt burden in low-income households triggers parental negative emotions, which subsequently damage children’s mental health. This is particularly critical in rural areas, where lower economic development forces families to borrow for basic needs, thereby exacerbating financial strain and child psychological distress. Demographically, we found that girls are more susceptible to depression linked to household debt, a finding that aligns with the work of Leve et al. (2005). Notably, there are discernible differences in how debt correlates with internalizing and externalizing behavioral problems based on the child’s sex. Girls appear to be more sensitive to fluctuations in the family environment, rendering them more vulnerable to psychological distress. In summary, higher household debt is consistently associated with a greater likelihood of psychological distress in children, with this effect being most acute among girls and children in socioeconomically disadvantaged households.

## 7. Conclusions and Implications

### 7.1. Conclusions

This study provides a detailed investigation into the relationship between household debt and children’s depressive symptoms in China, yielding key conclusions with valuable implications for policy. Our primary finding confirms Hypothesis 1: greater household debt is significantly associated with a higher risk of depressive symptoms among children. This positive association proves robust across a series of rigorous sensitivity tests.

This study also elucidates the mechanisms of this relationship. The link between household debt and children’s depressive symptoms is significantly mediated by a deterioration in parent–child relationships, specifically through reduced intimacy and increased conflict, thereby supporting Hypotheses 2a and 2b. Furthermore, we identified two key moderators that buffer this detrimental effect: the relationship is attenuated when fathers work longer hours to help repay the debt (supporting Hypothesis 3) and in households where parents report higher levels of marital satisfaction (supporting Hypothesis 5).

Our analysis further reveals significant heterogeneity in the effects of household debt. The detrimental impact on children’s mental health is primarily driven by housing debt and nonbank-sourced debt. Furthermore, this negative effect is disproportionately concentrated among vulnerable populations: it is significantly more pronounced in families with lower parental educational attainment and lower income levels, as well as among children living in rural China, where debt significantly elevates their risk of psychological distress.

### 7.2. Practical Implications

The findings of this study carry important implications for government, communities, schools, and households aiming to safeguard children’s mental health amid rising household debt:

First, to alleviate mortgage burdens among middle- and low-income households, the government should expand the supply of affordable housing through new construction, rehabilitation, and acquisition. Priority should be given to the housing needs of rural, middle- and low-income families, with an emphasis on increasing the availability of small, affordable units. Furthermore, minimum subsistence-allowance standards should be calibrated to rural residents’ per capita basic living expenses and indexed accordingly, ensuring a basic livelihood for households facing heavy housing costs and thereby mitigating the adverse effects of mortgage burdens on children’s mental health.

Second, community committee should strengthen community-level initiatives by establishing community-based service centers. Guided by the five action areas of the Ottawa Charter, these centers would serve as hubs for building healthy communities. A key initiative would be to deploy volunteer teams to regularly deliver financial courses for rural residents, including: focusing on family financial planning, debt restructuring, and financial risk prevention; Mental Health Literacy: covering emotion recognition, managing financial stress, and alleviating pressures related to marriage and child-rearing; Child Development & Parenting: teaching principles of child physical/psychological development, positive behavior guidance, effective parent–child communication, and strategies for providing emotional support. Moreover, the volunteer team can organise cultural and recreational activities to support children’s emotion regulation. These initiatives help bridge the relational gap between volunteers and children, providing a psychologically safe, low-pressure context in which children can articulate previously unexpressed emotions and reduce stress.

Third, to build foundational capabilities early, schools should integrate “future-parent” education into the middle-school curriculum. This would involve introducing financial literacy courses to impart essential knowledge and debt-risk prevention skills. Concurrently, foundational parenting courses should be offered to help adolescents understand child development and emotion regulation. A crucial component of this instruction is highlighting the mechanisms through which household economic pressure influences child outcomes specifically, by affecting the quality of parent–child interactions, which in turn shapes a child’s emotional and cognitive development. Furthermore, a school should hold regular parents’ meetings, undertake home visits, and conduct confidential one-to-one conversations to strengthen communication with families. This would enable staff to gain timely insight into children’s home circumstances and to support parents in managing stress, thereby reducing the risk of negative emotions being passed on to the children.

Fourth, to promote the development of a healthy family, family members should learn practical skills for managing debt-related stress. Parents should adopt proactive strategies that sustain marital harmony and a stable home environment, thereby strengthening children’s sense of security. Specifically, parents should be supported in using positive parenting practices and establishing effective communication routines that prevent the spillover of negative emotions to children during periods of financial strain, safeguarding children’s emotional regulation and cognitive development. Moreover, parents can help children develop positive strategies for expressing emotions, such as deep breathing, physical activity, and keeping a diary, enabling them to release negative feelings effectively.

## 8. Limitations

This study uses cross-sectional data, which limits causal interpretation between household debt and children’s depressive symptoms. Measurement of depressive symptoms relies on self-reported symptoms, potentially introducing bias. Unobserved factors may influence both debt and children’s depressive symptoms and reverse causality cannot be fully ruled out. Additionally, the findings based on Chinese data may have limited generalizability to other contexts. Lastly, more detailed categorization of debt types and objective measures of parent–child relationships could strengthen future research.

## Figures and Tables

**Table 1:** Descriptive Statistics and Definitions of Study Variables.

Variable	Definition	Obs	Mean	Std.Dev.	Min	Max
Explained variables						
Children’s depressive symptoms	CES-D 8 scores of children	805	4.061	3.368	0	19
Explanatory variables						
Household debt	Unpaid Debt of households	805	73,307	159,337	0	1,100,000
Mechanism variables						
Parent–child intimacy	Parent–child intimacy is measured by three items, items like “How often do you discuss school matters with your child?”, “How often do you require your child to complete his/her homework?” and “Do your parents know who you play with when you’re not at home?”	805	10.64	2.179	3	15
Parent–child conflict	Parent–child conflict is measured by the item: “How many times has the child quarreled with their parents in the past month?”	805	1.646	3.416	0	30
Father’s long working hours	Whether the working hours of a father exceed 44 h, assigned a value of 1, otherwise 0	805	0.868	0.338	0	1
Mother’s long working hours	Whether the working hours of a mother exceed 44 h, assigned a value of 1, otherwise 0	805	0.673	0.469	0	1
Marital satisfaction	Marital satisfaction is measured by the average value of both spouses’ self-reported satisfaction scores	805	4.426	0.691	1.500	5
Control variables						
Gender male	Gender of children	805	0.552	0.498	0	1
Educational attainment	Educational attainment of children	805	6.139	1.805	1	10
Health status	The number of times a child visited a doctor in the past year	805	0.343	0.475	0	1
Class rank	In the most recent major exam (midterm or final), the approximate class ranking of a child is categorized as follows: Top 10% = 5; 11–25% = 4; 26–50% = 3; 51–75% = 2; Bottom 24% = 1	805	3.021	1.729	0	5
Interpersonal relationships	Self-reported popularity: measured on a scale from 0 to 10, where 0 indicates the lowest level of popularity and 10 indicates the highest	805	6.948	2.001	1	10
Possession of a high school diploma	Whether either parent has completed high school education or above, assigned a value of 1, otherwise 0	805	0.379	0.485	0	1
Drinking behavior	Whether either parent has consumed alcohol more than three times per week in the past month, assigned a value of 1, otherwise 0	805	0.328	0.470	0	1
Household assets (Unit: CNY)	Household net assets	805	401,155	391,959	15,000	1,200,000
Car ownership	Whether the family owns a car, including in the forms of purchase and lease, assigned a value of 1, otherwise 0	805	0.508	0.500	0	1
Educational expenditures (Unit: CNY)	In the past 12 months, the family spent on education	805	6257	5343	800	15,000
Social interaction	In the past 12 months, the family spent on social interaction	805	3717	4406	0	20,000
Livelihood diversification	Livelihood diversification is measured by the Simpson Diversity Index	805	0.190	0.197	0	0.645
Regional location	Eastern region = 1; middle region = 2; western region = 3	805	1.898	0.824	1	3

Note: The data are obtained from two waves (2020 and 2022) of The China Family Panel Studies (CFPS).

**Table 2:** Benchmark Regression.

Variable	(1)	(2)
Ln (Household debt)	0.035 **	0.047 **
	(0.016)	(0.022)
Gender male		−0.363
		(0.246)
Educational attainment		0.006
		(0.067)
Health status		0.419 *
		(0.249)
Class rank		−0.133 *
		(0.072)
Interpersonal relationships		−0.275 ***
		(0.061)
Possession of a high school diploma		−0.010
		(0.248)
Drinking behavior		0.302
		(0.258)
Ln (Household assets)		−0.184 *
		(0.098)
Car ownership		−0.626 **
		(0.249)
Ln (Educational consumptions)		−0.314 ***
		(0.114)
Ln (Social interactions)		−0.073
		(0.062)
Livelihood diversification		1.181 **
		(0.600)
Regional location		0.130
		(0.154)
Constant	3.911 ***	11.320 ***
	(0.171)	(1.648)
Observations	805	805
R^2^	0.0028	0.0880
F	4.937	4.612

Note: Robust standard errors in parentheses. *, **, and *** are significant at levels of 10%, 5%, and 1%, respectively.

**Table 3:** Results of Robustness Test.

**Panel A: Estimate Results of 2SLS**						
			(1)			
Variable			IV-2SLS			
Ln (Household debt)			0.407 *			
			(0.244)			
Control Variables			Yes			
Observations			612			
F-value of the first stage			7.02			
T-value of instrumental variable			−2.88			
DWH Chi^2^/F-value			3.8463			
*p*-value			0.0503			
**Panel B: Estimate Results of PSM**						
Dependent variable	Matching methods	Experimental group (with debt)	Control group (no debt)	ATT	Standard error	*t*-value
Children’s depressive symptoms	Nearest neighbor matching (1:2)	4.0268	3.5435	0.4833 **	0.2409	2.01
	Nearest neighbor matching (1:4)	4.0268	3.5076	0.5193 **	0.2214	2.35
	radius matching (0.04)	4.0268	3.5997	0.4272 **	0.2065	2.07
	kernel matching (0.06)	4.0268	3.6008	0.4261 **	0.2063	2.06
**Panel C: Estimate Results of IPWRA**						
Outcome		Treatment effects				
Children’s depressive symptoms					Potential outcome mean (POM)	
		Debt			4.364 (0.222) **	
		Non-debt			3.876 (0.153) ***	
		Average treatment effect (ATE)			0.488	
		Percentage changes			11.18%	
		Observations			805	

Note: Robust standard errors in parentheses. *, **, and *** are significant at levels of 10%, 5%, and 1%, respectively.

**Table 4:** Replace Explanatory Variable (Debt Ratio).

	(1)	(2)
Variable	POLS	POLS
Debt-to-income	0.277 *	
	(0.158)	
Debt-to-asset		1.551 *
		(0.937)
Control variables	Yes	Yes
Observations	805	805
R^2^	0.087	0.087
F	4.582	4.521

Note: Robust standard errors in parentheses. * is significant at levels of 10%.

**Table 5:** Impact of Different Types of Debt on Children’s Depressive Symptoms.

Variable	(1)	(2)	(3)	(4)
Ln (Housing debt)	0.035 *			
	(0.017)			
Ln (Non-housing debt)		0.025		
		(0.022)		
Ln (Bank debt)			0.034	
			(0.023)	
Ln (Non-bank debt)				0.047 *
				(0.025)
Control variables	Yes	Yes	Yes	Yes
Observations	805	805	805	805
R^2^	0.086	0.084	0.086	0.087
F	19.88	18.71	4.560	4.385

Note: Robust standard errors in parentheses. * is significant at levels of 10%.

**Table 6:** Results of Heterogeneity Analysis.

**Panel A: The Impact of Children’s Gender**			
	(1)	(2)	
Variable	girl	boy	
Ln (Household debt)	0.095 ***	0.023	
	(0.037)	(0.028)	
Control variables	Yes	Yes	
Observations	361	444	
R^2^	0.138	0.090	
F	3.668	3.114	
**Panel B: The Impact of Parental Education Attainment**			
	(1)	(2)	(3)
Variable	Primary school and below	High school	Tertiary education
Ln (Household debt)	0.042 **	0.066	0.079
	(0.017)	(0.049)	(0.073)
Control variables	Yes	Yes	
Observations	500	168	137
R^2^	0.087	0.151	0.154
F	19.00	2.324	20.20
**Panel C: The Impact of Different Income Level**			
	(1)	(2)	(3)
Variable	Low income	Middle income	High income
Ln (Household debt)	0.064 **	−0.005	0.067
	(0.030)	(0.028)	(0.042)
Control variables	Yes	Yes	Yes
Observations	269	269	267
R^2^	0.151	0.122	0.067
F	48.26	4.602	0.945
**Panel D: The Impact of Different Regions**			
	(1)	(2)	
Variable	Rural areas	Urban areas	
Ln (Household debt)	0.051 *	0.044	
	(0.030)	(0.033)	
Control variables	Yes	Yes	
Observations	424	381	
R^2^	0.095	0.138	
F	2.719	5.416	

Note: Robust standard errors in parentheses. *, **, and *** are significant at levels of 10%, 5%, and 1%, respectively.

**Table 7:** Result of the Mediating Effect of Parent–Child Relationship.

**Panel A: The Mediating Effect of Parent–Child Intimacy**			
	(1)	(2)	(3)
	Children’s depressive symptoms	Parent–child intimacy	Children’s depressive symptoms
Ln (Household debt)	0.047 **	−0.026 *	0.043 *
	(0.022)	(0.015)	(0.023)
Parent–child intimacy			−0.156 ***
			(0.058)
Control	YES	YES	YES
Observations	805	805	805
R^2^	0.088	0.093	0.097
F	4.612	5.685	5.161
**Panel B: The Mediating Effect of Parent–Child Conflict**			
	(1)	(2)	(3)
	Children’s depressive symptoms	Parent–child conflict	Children’s depressive symptoms
Ln (Household debt)	0.047 **	0.059 **	0.036 *
	(0.022)	(0.024)	(0.021)
Parent–child conflict			0.178 ***
			(0.050)
Control	YES	YES	YES
Observations	805	805	805
R^2^	0.088	0.030	0.120
F	4.612	1.223	5.457

Note: Robust standard errors in parentheses. *, **, and *** are significant at levels of 10%, 5%, and 1%, respectively.

**Table 8:** Result of the Moderating Effect of Long Working Hours.

Variable	(1)	(2)	(3)	(4)	(5)
Ln (Household debt)	0.150 **	0.005	0.148 **	0.032	0.048 **
	(0.064)	(0.035)	(0.063)	(0.038)	(0.023)
Father’s long working hours	0.324				
	(0.467)				
Ln (Household debt) × Father’s long working hours	−0.118 *				
	(0.069)				
Mother’s long working hours		−0.137			
		(0.310)			
Ln (Household debt) × Mother’s long working hours		0.065			
		(0.044)			
Father’s long working hours (40 h)			0.396		
			(0.448)		
Ln (Household debt) × Father’s long working hours (40 h)			−0.116 *		
			(0.068)		
Mother’s long working hours (40 h)				0.056	
				(0.323)	
Ln (Household debt) × Mother’s long working hours (40 h)				0.024	
				(0.047)	
Marital satisfaction					−0.340 **
					(0.159)
Ln (Household debt) × Marital satisfaction					−0.054 *
					(0.031)
Control variable	Yes	Yes	Yes	Yes	Yes
Observations	805	805	805	805	805
R^2^	0.092	0.091	0.092	0.089	0.096
F	4.197	4.099	4.206	4.071	4.30

Note: Robust standard errors in parentheses. * and ** are significant at levels of 10% and 5%, respectively.

## Data Availability

The data used and analyzed during the current study are available from the China Family Panel Studies (CFPS) (URL: https://www.isss.pku.edu.cn/cfps/, accessed on 3 November 2024).
